# The naringenin-dependent regulator FdeR can be applied as a NIMPLY gate controlled by naringenin and arabinose

**DOI:** 10.1093/synbio/ysae001

**Published:** 2024-01-16

**Authors:** Fernanda Miyuki Kashiwagi, Brenno Wendler Miranda, Emanuel Maltempi de Souza, Marcelo Müller-Santos

**Affiliations:** Postgraduate Program in Science (Biochemistry), Department of Biochemistry and Molecular Biology, Nitrogen Fixation Laboratory, Federal University of Paraná (UFPR), Curitiba, Brazil; Postgraduate Program in Science (Biochemistry), Department of Biochemistry and Molecular Biology, Nitrogen Fixation Laboratory, Federal University of Paraná (UFPR), Curitiba, Brazil; Postgraduate Program in Science (Biochemistry), Department of Biochemistry and Molecular Biology, Nitrogen Fixation Laboratory, Federal University of Paraná (UFPR), Curitiba, Brazil; Postgraduate Program in Science (Biochemistry), Department of Biochemistry and Molecular Biology, Nitrogen Fixation Laboratory, Federal University of Paraná (UFPR), Curitiba, Brazil

**Keywords:** FdeR, naringenin, P*_BAD_*, arabinose, NIMPLY gate

## Abstract

The FdeR regulator has been reported as a transcriptional activator dependent on the interaction with naringenin. Previously, FdeR and its cognate promoter were used to construct naringenin-sensitive sensors, though no correlation was associated between the FdeR level of expression and outputs. Therefore, to understand this correlation, we constructed a circuit with FdeR expression adjusted by the arabinose concentration through an AraC-P*_BAD_* system and the FdeR-regulated promoter controlling the expression of GFP. We observed a significant reduction in the activity of the target promoter by increasing FdeR expression, indicating that although FdeR has been primarily classified as a transcriptional activator, it also represses transcription. Leveraging the bifunctional feature of FdeR, acting as both transcriptional activator and repressor, we demonstrated that this genetic circuit, when previously switched on by naringenin, can be switched off by inducing an increased FdeR expression level. This engineered system functioned as a NIMPLY gate, effectively decreasing GFP expression by 50% when arabinose was added without removing naringenin from the medium. Exploiting FdeR versatility, this study demonstrates an innovative application of this transcriptional factor for developing novel NIMPLY gates activated by a molecule with low toxicity and nutraceutical properties that may be important for several applications.

**Graphical Abstract**

**Figure:**
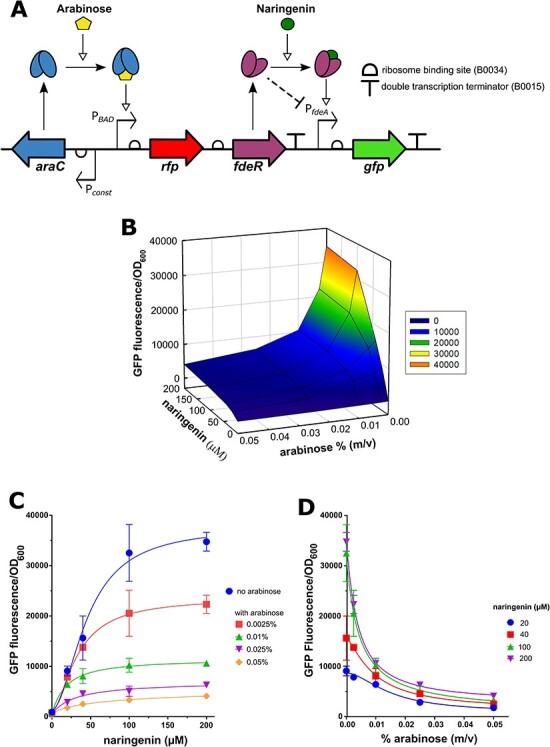

## 1. Introduction

The main target of synthetic biology is to program a living cell to give a desired response to environmental or cellular signals. To achieve this purpose, genetic circuits of varying complexity have been constructed for different applications, such as medicine, the bioproduction of chemicals and environmental sensing (1–3).

There are some examples of genetic circuits that respond to metabolites secreted by plants (4–6), such as flavonoids. Flavonoids are low-molecular weight specialized metabolites of plants and play an important role as specific transmitters in some symbiotic relations with bacteria (7). In rhizobia, flavonoids modulate the expression of *nod* genes involved in legume nodule formation during nitrogen-fixing endosymbiosis (8). Although the role of flavonoids remains elusive for other plant-growth-promoting bacteria, some rhizobacteria might use flavonoids as a carbon source or degrade them to avoid their toxic effects, giving them a competitive advantage over other rhizobacteria (9).

The plant-associative bacterium *Herbaspirillum seropedicae* possesses a naringenin degradation pathway, capable of using it as the sole carbon source. The *fde* (flavonoid degradation) operon is regulated by FdeR, a LysR-type transcriptional regulator (LTTR) (10, 11). The intergenic region between *fdeR* and *fdeA* (the first gene of the *fde* operon) contains 14 potential LTTR boxes for the binding of FdeR (T-N_11_-A sequence) from which nine of them are clustered in a 50-bp region, and eight overlapped the −35 site and UP element of the σ70 promoter P*_fdeA_* (12). DNase I footprinting showed that FdeR protected a 60-bp region in the intergenic region between *fdeR* and *fdeA*. Moreover, the binding of flavonoids such as naringenin, chrysin and daidzein to DNA-bound FdeR keeps FdeR bound in the P*_fdeA_*. Still, it induces topological changes in the complex, which facilitates the occupancy of the P*_fdeA_* by the RNA polymerase (12). The *fdeR*^−^ mutant of *H. seropedicae* does not express the *fde* operon and, therefore, has a significant reduction in the degradation of naringenin in culture, corroborating the role of FdeR as a transcriptional activator in the organism of origin (10).

Previous studies applied the FdeR system of *H. seropedicae* to construct biosensors detecting naringenin in *Escherichia coli* (13–15). Although the expression level of either the transcriptional factor FdeR or the reporter gene has already been regulated through different synthetic ribosome-binding sites (RBS), no correlation could be established to explain the variation in the response curves (14).

In this study, we investigated the effect of the expression level of FdeR on the modulation of the response curves by constructing a genetic circuit with *fdeR* expression controlled by an arabinose-inducible promoter. We demonstrated that FdeR behaves as both a transcriptional activator and a repressor, and showed that combined with the AraC-P*_BAD_* system, the output of the genetic circuit is only switched on in the presence of naringenin and absence of arabinose, acting as a NIMPLY logic gate. Moreover, when the genetic circuit is previously induced by naringenin, it is turned off by simply increasing FdeR expression levels.

## 2. Materials and methods

### 2.1 Bacterial strains and growth conditions

*Escherichia coli* TOP10 (Invitrogen, USA) was used for cloning purposes, and *E. coli* MG1655 was used to test the genetic circuits. *Escherichia coli* were grown in lysogeny broth (LB) (16) at 37°C and shaking at 160 rpm. Antibiotics were added at the following concentrations: streptomycin (80 µg/ml) and chloramphenicol (25 µg/ml).

### 2.2 Plasmids construction

The plasmids were first constructed on the backbone of pSB1C3 following the BioBrick assembly method (17). The following parts were obtained from the Registry of Standard Biological Parts: constitutive promoters (BBa_J23110 and BBa_J23114), RBS (BBa_B0034), transcription terminator (BBa_B0015), the composite part formed by an RBS, *gfp* and transcription terminator (BBa_I13504), RBS and *rfp* (BBa_I13502) and the composite part containing promoter P*_BAD_* and the repressor AraC (BBa_K808000). The *fdeR* gene was synthesized on demand (Genscript, USA), identical to the native sequence from *H. seropedicae*, except for one nucleotide change to remove an EcoRI site. The *fdeR–fdeA* intergenic region, hereafter referred to as P*_fdeA_*, was PCR amplified from *H. seropedicae* SmR1 genomic DNA. Bacterial strains and plasmids are listed in Table 1.

**Table 1.** Bacterial strains and plasmids used in this work

Bacterial strains	Relevant characteristics	Reference
*E. coli* TOP10	Cloning strain	Invitrogen
*E. coli* MG1655	*E. coli* K-12-derivative strain	(18)
**Plasmids**	**Relevant characteristics**	**Reference**
pSB1C3	High-copy number plasmid for Biobricks assembly, pMB1 origin of replication, Cm^R^	iGEM repository
pFMK13	Cm^R^, *araC*-P*_BAD_*-RBS-*rfp-fdeR*-T-P*_fdeA_*-RBS-*gfp*-T assembled in pSB1C3	This work

RBS refers to the B0034 BioBrick code; T refers to the B0015 BioBrick code; *gfp* is the gene expressing the GFPmut3b variant of GFP, *rfp* is the gene expressing mRFP1 a variant of DsRed.

### 2.3 Cell fluorescence measurement

A single colony of *E. coli* MG1655 carrying the plasmid pSB1C3-*araC*-P*_BAD_-rfp-fdeR*-P*_fdeA_-gfp* (pFMK13) was cultured overnight in liquid LB at 37°C and 160 rpm. These cultures were diluted (1:100) in 200 µl of fresh LB with different concentrations of arabinose and incubated in 96-well plates (Greiner Bio-one, 96 Flat Clear Bottom Black Polystyrene) until reaching optical density at 600 nm (OD_600_) of ∼0.7. Different concentrations of naringenin dissolved in dimethyl sulfoxide were added. The culture was incubated for 6 h, with shaking at 160 rpm. The GFP and RFP emitted fluorescence, and OD_600_ was measured on Synergy LX (Biotek, USA). GFP fluorescence was measured with excitation at 485 nm and emission at 528 nm; RFP fluorescence was measured with excitation at 530 nm and emission at 590 nm.

An overnight culture of *E. coli* MG1655 containing the pFMK13 was diluted 100× in 60 ml penicillin glass flasks containing 10 ml of LB medium to determine the switch-off. They were then incubated in a shaker at 37ºC and 180 rpm. After 3 h, naringenin was added to the cultures to 100 µM final concentration. After 3 h of induction, the cultures were washed twice with LB medium, 200 µl distributed in each well of a 96-well plate and incubated at 37ºC with 5 mm orbital shaking in a microplate reader Tecan Infinite 200 (Switzerland) for 270 min. After washing, the cultures were resuspended with LB medium supplemented with arabinose and naringenin. The fluorescence was recorded every 10 min.

### 2.4 Hill fitting

The experimental data of normalized GFP fluorescence (GFP fluorescence/OD_600_) was fitted with the Hill function as follows:


$$\frac{{GFP\,fluorescence}}{{O{D_{600}}}} = {y_0} + \beta \frac{{Na{r^n}}}{{(Na{r^n} + K_{0.5}^n)}}$$

with *y_0_* = basal normalized fluorescence, *β* = relative maximum normalized fluorescence, *Nar =* concentration of naringenin in µM, *n *= Hill coefficient, and *K_0.5_* = Hill constant (half-maximal naringenin concentration, µM).

The experimental data of normalized GFP fluorescence (GFP fluorescence/OD_600_) repressed by arabinose addition was fitted with the Hill function as follows:


$$\frac{{GFP\,fluorescence}}{{O{D_{600}}}} = {y_0} + \beta \frac{{K_{0.5}^n}}{{(Ar{a^n} + K_{0.5}^n)}}$$

with, *y_0_* = minimum normalized fluorescence, *β* = maximum normalized fluorescence, *Ara =* concentration of arabinose in %, *n *= Hill coefficient, and *K_0.5_* = Hill constant (half-maximal arabinose concentration, %).

## 3. Results

### 3.1 High expression of FdeR represses the *fdeA* promoter

In our previous study, we obtained different output responses for genetic circuits by solely varying the expression level of the transcriptional factor, using constitutive promoters with different strengths (19). Here, aiming to understand the impact of the expression level of the transcriptional factor FdeR, we constructed an arabinose inducible system to control *fdeR* expression.

**Figure 1.**
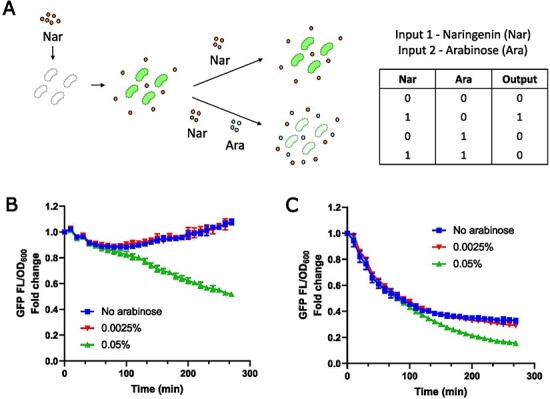
FdeR transcriptional regulator acts as both an activator and a repressor. (A) Scheme of the genetic circuit showing the *araC* gene expressing the AraC regulator, which activates the transcription of the RFP reporter and the FdeR transcriptional regulator in the presence of arabinose. FdeR, in turn, in the presence of the flavonoid naringenin, activates the expression of the GFP reporter. Note that the dashed arrow shows the repressor effect of FdeR not bound to naringenin on the P*_fdeA_* promoter. (B) A surface plot correlating naringenin and arabinose concentrations was added to the culture medium, and GFP fluorescence/OD_600_ was expressed as the output on the *y*-axis. When arabinose was added to a final concentration of 0.05%, even 200 µM of naringenin could not induce high GFP expression. (C–D) plots of fluorescence versus inducer concentration (naringenin and arabinose, respectively), showing the circuit switch-off at high arabinose concentration. Each response curve was adjusted with the Hill equation as an activating input (C) or repressing input (D). Note that arabinose concentrations between 0.01% and 0.05% lead to an abrupt switch-off in GFP expression. Mean values and standard deviations from three replicates are shown. Standard deviations cannot be seen on graphs at some points because the distance between the error bars is smaller than the symbol size.

We considered the gene coding for the transcriptional factor FdeR of *H. seropedicae* and the promoter of the *fdeA* gene (P*_fdeA_*), which FdeR binds to, as two independent genetic parts. We associated them with an RBS, a transcription terminator, reporter genes and an arabinose-inducible regulatory unit (AraC-P*_BAD_*). The *fdeR* gene was controlled by the arabinose-inducible P*_BAD_* promoter, which is activated by the transcriptional factor AraC bound to arabinose. To evaluate the activity of P*_BAD_*, we added the reporter gene *rfp* in an operon together with *fdeR*, and to monitor the P*_fdeA_* activity, the reporter gene *gfp* was used (Figure 1A).

To express FdeR at different levels, arabinose was added to concentrations between 0% and 0.05% to the culture of *E. coli* MG1655 carrying the construction *araC*-P*_BAD_-rfp-fdeR*-P*_fdeA_-gfp*. After 3 h from arabinose addition, the P*_BAD_* promoter was active, reaching its maximum values of RFP fluorescence between 0.025% and 0.05% arabinose (Supplementary Figure S1). Hence, we assumed that at this stage, FdeR levels varied according to arabinose concentration, and we induced them with naringenin.

Besides indirectly monitoring the FdeR expression, RFP expression also checks the cross-reactivity of naringenin into the AraC-P*_BAD_* system. At 9 h of cultivation (6 h after induction with naringenin), RFP fluorescence mainly depended on the concentration of arabinose (Supplementary Figure S2A). In the absence and in the lowest concentration of arabinose (0.0025%), there was a slight cross-reactivity of naringenin in P*_BAD_* activity (Supplementary Figure S2B). However, such perturbation was mainly restricted to very low arabinose concentration. Therefore, this minimal cross-reactivity would not significantly impact our final objective of evaluating the correlation between FdeR level and P*_fdeA_* activity.

The activity of the P*_fdeA_* promoter, measured through GFP fluorescence, was dependent on both arabinose and naringenin concentrations, i.e. the level of FdeR and its cognate inducer concentration (Figure 1B). For each concentration of arabinose, including in its absence, naringenin-induced GFP expression (Figure 1C and D), and the normalized GFP fluorescence/OD_600_ could be fitted with the Hill equation (Supplementary Table S1). Without arabinose, the promoter P*_BAD_* had a leaky *fdeR* transcription, enough to induce P*_fdeA_* when adding naringenin. The increase in arabinose concentration decreased basal output and maximum output, showing that an increase in FdeR levels represses P*_fdeA_*.

Therefore, we also analyzed the data by fixing each concentration of naringenin (Figure 1D), in other words, with arabinose as the input. The Hill equation could fit the response curves as a repressing input (Supplementary Table S2). To sum up, we demonstrated that high levels of FdeR repress P*_fdeA_*, even at inducing naringenin concentrations (i.e. 200 µM).

It is worth noting that without *fdeR* expression, P*_fdeA_* showed a low basal expression regardless of the naringenin concentration (Supplementary Figure S3), proving that FdeR acts primarily as a transcriptional activator of *fdeA,* as previously reported (10, 12, 14). To further validate that higher FdeR levels reduce P*_fdeA_* activity, *fdeR* was expressed under the control of two constitutive promoters with different transcription strengths. When a low-strength constitutive promoter (J23114) controlled *fdeR*, we activated the circuit with increasing concentrations of naringenin, reaching 40-fold the output of P*_fdeA_-gfp* with 200 µM naringenin (Supplementary Figure S3 and Supplementary Table S3). By increasing *fdeR* expression through the constitutive promoter J23110, with 200 µM naringenin, the output was only 3-fold above that of P*_fdeA_-gfp*. These results demonstrate that although FdeR is primarily a transcriptional activator, it also acts as a repressor at high expression levels.

### 3.2 The circuit is switched off by arabinose, even in high concentration of naringenin

**Figure 2.**
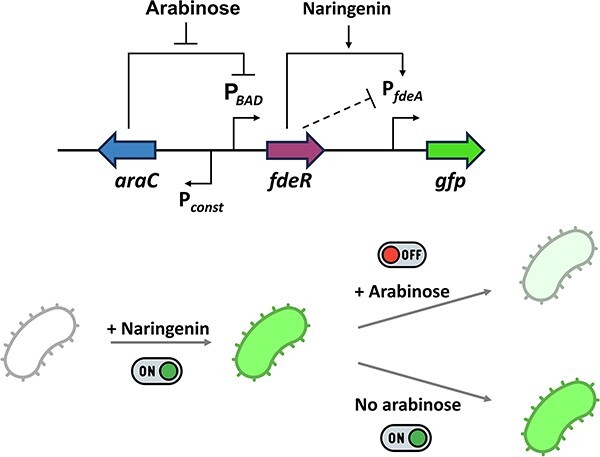
Gene expression is switched off even in the presence of high naringenin concentration. (A) *Escherichia coli* MG1655 cultures carrying plasmi 0d pFMK13 were grown in LB medium, previously induced with 100 µM naringenin. The cells were resuspended in LB medium containing 100 µM naringenin, and different concentrations of arabinose were tested. The truth table on the side demonstrates the logic following the NIMPLY rules. (B) GFP fluorescence was monitored over 270 min after arabinose addition. The *y*-axis of the graph represents GFP fluorescence/OD_600_ fold change to 0 min from arabinose addition. (C) *Escherichia coli* cells, previously induced with 100 µM naringenin, were resuspended in LB medium without naringenin and different concentrations of arabinose were added. GFP fluorescence was monitored over 270 min after arabinose addition. Mean values and standard deviations from three replicates are shown. Standard deviations cannot be seen on graphs at some points because the distance between the error bars is smaller than the symbol size.

To exploit the dual role of FdeR as an activator and repressor, we applied the genetic circuit dependent on arabinose and naringenin to function as a NIMPLY gate. We followed fluorescence over time in cultures previously induced with naringenin and challenged with arabinose (Figure 2A and B). Adding 0.0025% arabinose was likely insufficient to switch off the circuit, as it showed a similar GFP expression to the condition without arabinose, with a slight reduction up to 60 min and reaching the initial fluorescence at 230 min. On the other hand, 0.05% arabinose, even in the presence of high naringenin concentration (100 µM), continuously dropped GFP expression, reducing the initial fluorescence by 50% after 270 min. Therefore, we demonstrate that this genetic circuit acts as a NIMPLY logic gate, in which the output is switched on only when input 1 (naringenin) is present and input 2 (arabinose) is absent.

We also tested a hypothetical situation when naringenin is removed from the medium and checked if a further decrease in gene expression is obtained (Figure 2C). Cultures previously grown with 100 µM naringenin were washed to remove naringenin. Adding 0.05% arabinose, the reduction of GFP expression was sharper, around 85% after 270 min, compared to around 70% without arabinose or 0.0025% arabinose. Therefore, although naringenin removal drops P*_fdeA_* activity, an enhanced repressor effect can be achieved when more FdeR is expressed.

## 4. Discussion

Our study demonstrated that the transcription factor FdeR, responsive to the flavonoid naringenin, exhibits dual functionality as both an activator and a repressor. Leveraging this property, we have successfully constructed a NIMPLY logic gate. Furthermore, we have conceived a method to deactivate the circuit by increasing FdeR expression through an additional input, thereby rendering it a potential tool for applications that necessitate temporary gene expression, such as in metabolic engineering, circumventing unnecessary energy consumption. Additionally, we have explored situations where naringenin may be removed from the medium, such as through degradation or dilution, and instances where its removal is not feasible, such as in a batch culture.

One potential explanation for the repressive effect of FdeR is that an abundance of this transcription factor, when bound to DNA, could potentially occlude the −35 region of P*_fdeA_*, which becomes exposed in the presence of naringenin. Alternatively, elevated expression of FdeR could lead to an increased pool of free FdeR, which might titrate naringenin, as free FdeR has an affinity for binding to naringenin (12). As a result, DNA-bound FdeR and free FdeR may need to compete for naringenin molecules. Our results show that free FdeR likely has an advantage in finding and binding to naringenin in the cytosol compared to FdeR, which is bound to DNA.

FdeR ability to function as both an activator and a repressor streamlines the construction of intricate genetic devices. In a previous study, a NIMPLY genetic gate relied on three transcription factors and the modification of promoters by adding repressor sites (20), which significantly increased the workload required to characterize and enhance a genetic circuit. CRISPRi has been utilized as another tool in constructing NIMPLY logic gates (21), but it is important to note that dCas9 may potentially induce unforeseen burden effects on the organism due to off-target activity (22).

Given that naringenin is a metabolite released by plants, we anticipate that FdeR could find application in plant–microbe interactions. Similar to a prior study that showed bacterial N_2_-fixation only in the presence of signaling molecules exuded by plants (6), naringenin could be an inducer to regulate bacterial gene expression when in proximity to plants. Furthermore, due to naringenin nutraceutical properties and low toxicity, FdeR could be extended to various applications, including drug delivery and microbiome engineering. Lastly, we believe that alternative expression systems governing FdeR expression levels, apart from AraC-P_BAD_, are highly likely to uphold the NIMPLY logic response, potentially broadening the scope of applications, provided there is no cross-reactivity between the inducer molecules.

## Supplementary Material

ysae001_Supp

## Data Availability

Supplementary Data is available at SYNBIO online.

## Material availability statement

The plasmids constructed for this work are available on request to the corresponding author.
