# Supplementary material for: The naringenin-dependent regulator FdeR can be applied as a NIMPLY gate controlled by naringenin and arabinose
Source: Synth Biol (Oxf). 2024 Jan 16;9(1):ysae001. doi: 10.1093/synbio/ysae001 (PMC10799723; doi:10.1093/synbio/ysae001)
Supplement: ysae001_Supp [file ysae001_supp.zip › Supplementary Data_After revision_Final version.docx]

**Supplementary data**

**The naringenin-dependent regulator FdeR can be applied as a NIMPLY gate controlled by naringenin and arabinose**

**Fernanda Miyuki Kashiwagi^1^, Brenno Wendler Miranda^1^, Emanuel Maltempi de Souza^1^, Marcelo Müller-Santos^1*^**

^1^Postgraduate Program in Science (Biochemistry), Department of Biochemistry and Molecular Biology, Nitrogen Fixation Laboratory, Federal University of Paraná (UFPR), Curitiba, Brazil.

* Corresponding author: Marcelo Müller-Santos

e-mail address: [marcelomuller@ufpr.br](mailto:marcelomuller@ufpr.br)

**Supplementary Table 1.** Parameters for Hill fitting equation for the experimental data of the genetic circuit *araC*-P*_BAD_*-*fdeR*-P*_fdeA_* activated by naringenin.

| **[Arabinose], %** | ***y_0_*** | ***β*** | ***n*** | ***K_0.5_* (µM)** | ***R^2^*** |
| --- | --- | --- | --- | --- | --- |
| 0 | 1,657 | 36,484 | 1.89 | 47.01 | 0.94 |
| 0.0025 | 807.3 | 22,736 | 1.64 | 32.94 | 0.94 |
| 0.01 | 677.5 | 10,826 | 1.05 | 18.31 | 0.95 |
| 0.025 | 467.3 | 6,508 | 1.00 | 30.57 | 0.92 |
| 0.05 | 363.1 | 5,398 | 0.77 | 73.2 | 0.99 |

y_0_, basal output; β, maximum output; K_0.5_, naringenin concentration to reach half of maximum output; n, Hill coefficient; R^2^, coefficient of determination.

**Supplementary Table 2.** Parameters for Hill fitting equation for the experimental data of the genetic circuit *araC*-P*_BAD_*-*fdeR*-P*_fdeA_* repressed by arabinose.

| **[naringenin], µM** | ***y_0_*** | ***β*** | ***n*** | ***K_0.5_* (%)** | ***R^2^*** |
| --- | --- | --- | --- | --- | --- |
| 20 | 816.9 | 7,876 | 1.78 | 0.015 | 0.96 |
| 40 | 1,217 | 14,454 | 1.34 | 0.009 | 0.89 |
| 100 | 684.7 | 31,826 | 1.00 | 0.004 | 0.94 |
| 200 | 2,553 | 32,197 | 1.11 | 0.004 | 0.99 |

y_0_, minimum output; β, maximum output; K_0.5_, arabinose concentration to reach half of maximum output; n, Hill coefficient; R^2^, coefficient of determination.

**Supplementary Table 3.** Parameters for Hill fitting equation for the experimental data of the genetic circuit 110-*fdeR*-P*_fdeA_* and 114-*fdeR*-P*_fdeA_*.

| **Genetic circuit** | **y_0_** | **β** | **n** | **K_0.5_ (µM)** | **R^2^** |
| --- | --- | --- | --- | --- | --- |
| 110-*fdeR*-P*_fdeA_* | 109.3 | 1,224 | 1.76 | 87.08 | 0.99 |
| 114-*fdeR*-P*_fdeA_* | 336.0 | 15,951 | 2.01 | 44.74 | 0.99 |

y_0_, minimum output; β, maximum output; K_0.5_, arabinose concentration to reach half of maximum output; n, Hill coefficient; R^2^, coefficient of determination.

**Supplementary Figure 1.** Induction of RFP expression by the AraC-arabinose/P_BAD_ regulatory system.

*E. coli* MG1655 was transformed with the pFMK13. Cells were grown in LB medium with different concentrations of arabinose on a 96-well plate. At OD_600_ of approximately 0.7 (after 3 hours of induction with arabinose), the RFP fluorescence and OD_600_ were measured at Synergy LX (Biotek, USA). The graph shows the RFP fluorescence normalized by OD_600_ in arbitrary units. The black circles represent the means, and the error bars are the standard deviations from three replicates.


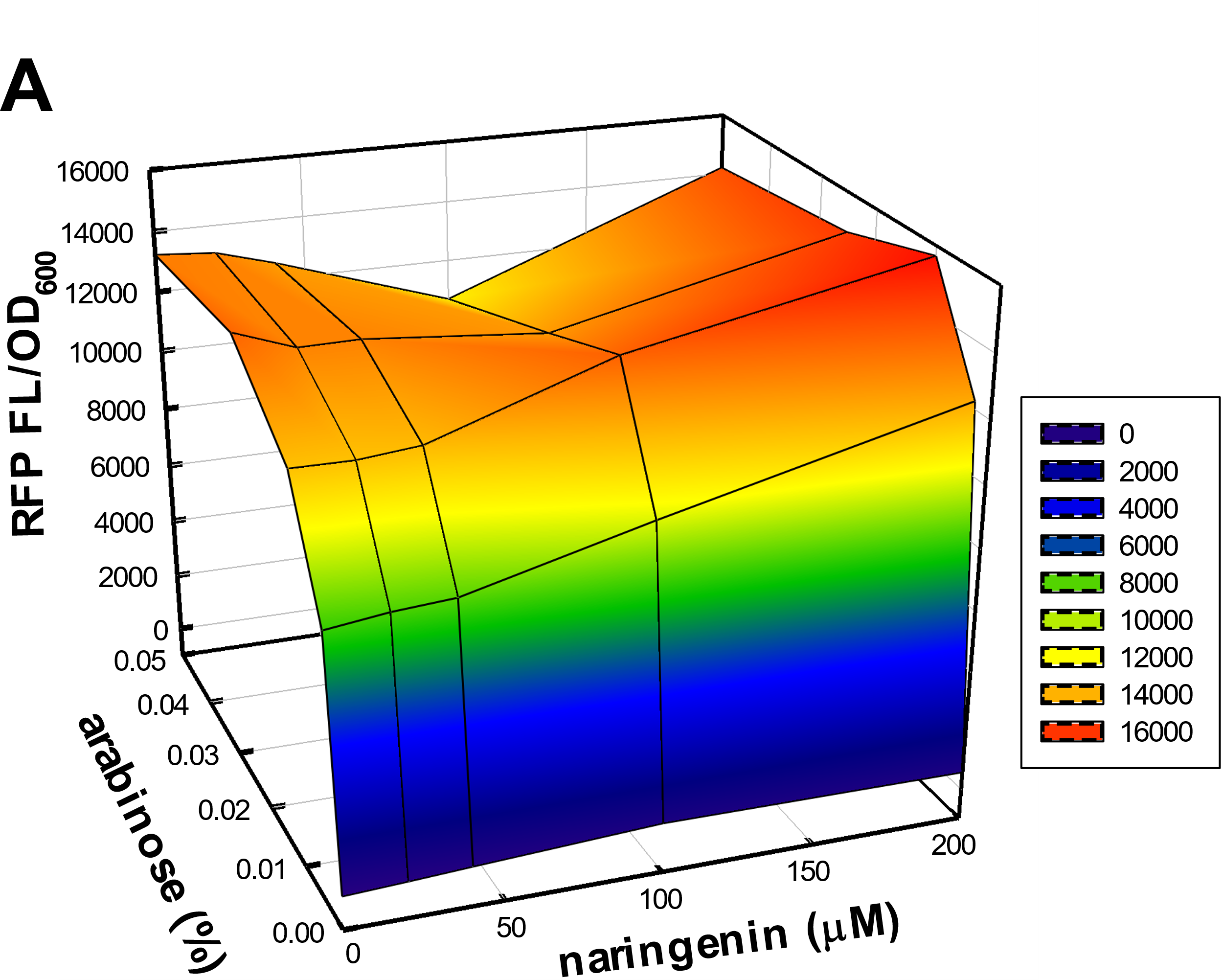
**Supplementary Figure 2.** Induction of RFP expression as a function of arabinose and naringenin concentrations

**(A)** Surface plot correlating naringenin and arabinose concentrations added to the culture medium and RFP fluorescence expressed as the output on the y-axis. When arabinose was added to a final concentration of 0.05%, a high RFP expression was achieved in all naringenin concentrations, showing that P*_BAD_* activated *fdeR* expression mediated by the quantity of arabinose but independent of naringenin in the medium. **(B)** Plots of RFP fluorescence versus naringenin concentration showing the circuit expressed constant RFP independent of the naringenin added to the medium. Symbols represent mean values and error bars represent the standard deviation from three replicates. At some points, standard deviations cannot be seen on graphs because the distance between the error bars is smaller than the symbol size.

**Supplementary Figure 3.** Modulation of FdeR levels can generate different outputs.


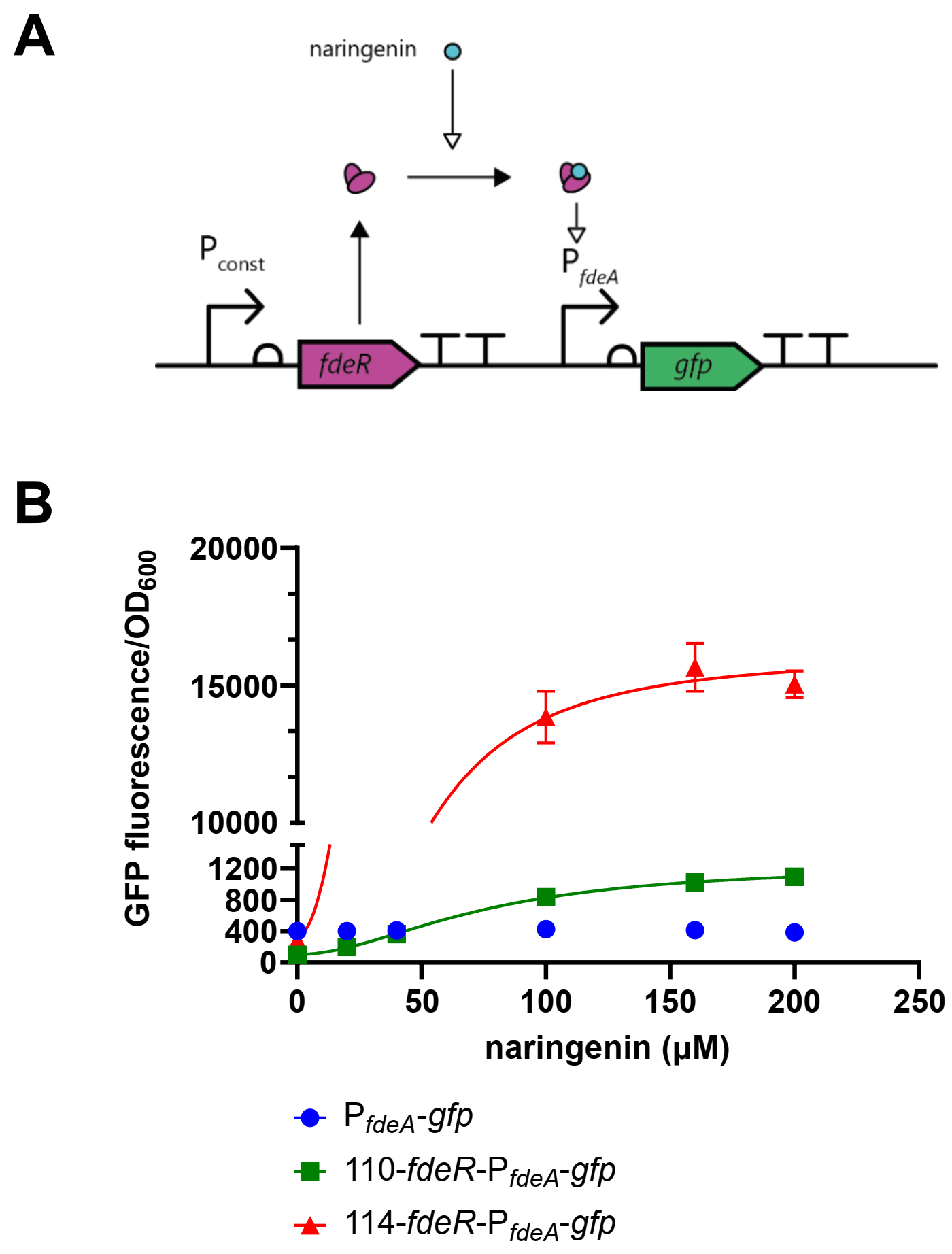


**(A)** Scheme of the genetic circuits with FdeR level of expression controlled by different constitutive promoters. 110-*fdeR*-P*_fdeA_*-*gfp* and 114-*fdeR*-P*_fdeA_*-*gfp* represent genetic circuits with a constitutive promoter (J23110 or J23114, respectively) controlling gene expression of *fdeR* gene and the P*_fdeA_* regulating *gfp* expression. As reported in our previous work, J23114 has 37 times lower transcription strength in comparison to J23110 (Kashiwagi et al., 2021). (**B)** Response curves for two genetic circuits with *fdeR* under the control of constitutive promoters. P*_fdeA_*-*gfp* represents only the sensor module, without the expression of *fdeR*. *E. coli* MG1655 was transformed with a pUC19-derived pMB1 origin of replication plasmid carrying one of the genetic circuits (110-*fdeR*-P*_fdeA_*, 114-*fdeR*-P*_fdeA_*). Cells were grown on a 96-well plate, at OD_600_ of approximately 0.7, and different concentrations of naringenin were added. The GFP fluorescence and OD_600_ were measured at Tecan Infinite 200 plate reader, after 6 hours of induction. The graph shows the GFP fluorescence normalized by OD_600_ in arbitrary units. The experimental data were fitted by the Hill equation (parameters provided in Supplementary Table 4), which is represented by the continuous lines. Symbols represent mean values and error bars represent the standard deviation from three replicates. At some points, standard deviations cannot be seen on graphs because the distance between the error bars is smaller than the symbol size.
